# Saliva as an alternative specimen for detection of Schmallenberg virus-specific antibodies in bovines

**DOI:** 10.1186/s12917-015-0552-0

**Published:** 2015-09-15

**Authors:** Justas Lazutka, Aliona Spakova, Vilimas Sereika, Raimundas Lelesius, Kestutis Sasnauskas, Rasa Petraityte-Burneikiene

**Affiliations:** Institute of Biotechnology, Vilnius University, V. A. Graiciuno 8, 02241 Vilnius, Lithuania; Institute of Microbiology and Virology, Veterinary Academy, Lithuanian University of Health Sciences, Tilzes 18, 47181 Kaunas, Lithuania

**Keywords:** Schmallenberg virus, Indirect ELISA, Antibody detection, Cattle, Serum, Milk, Saliva

## Abstract

**Background:**

Schmallenberg virus (SBV), discovered in continental Europe in late 2011, causes mild clinical signs in adult ruminants, including diarrhoea and reduced milk yield. However, fetal infection can lead to severe malformation in newborn offspring. Enzyme-linked immunosorbent assays (ELISA) are commercially available for detection of SBV-specific antibodies in bovine sera and milk. Here we describe the development and evaluation of an indirect ELISA based on a yeast derived recombinant SBV nucleocapsid protein (N) for the detection of SBV-specific antibodies in bovine saliva. Development of a non-invasive test to detect antibodies in individual bovine saliva samples could potentially provide a test suitable for calves and adult cattle. The aim of this study was to investigate the agreement between the levels of antibodies (IgG) measured in milk and sera, and the level of antibodies (IgG and IgA) in saliva, in comparison with the antibody levels detected in sera and milk with commercially available test.

**Results:**

Serum, milk and saliva samples from 58 cows were collected from three dairy herds in Lithuania and tested for the presence of SBV-specific antibodies. The presence of IgG antibodies was tested in parallel serum and milk samples, while the presence of IgA and IgG antibodies was tested in saliva samples. The presence of SBV-specific IgG and IgA in saliva was tested using an indirect ELISA based on a yeast-derived recombinant N protein. The presence of SBV-specific IgG in milk and sera was tested in parallel using a commercial recombinant protein based test. The sensitivities of the newly developed tests were as follows: 96 % for the IgG serum assay and 94 % for the IgG milk assay and 85 % and 98 % for IgG and IgA in saliva tests, when compared with data generated by a commercial IgG assay.

**Conclusions:**

Data from testing the saliva IgG and IgA and also the milk and serum IgG with indirect SBV-specific ELISAs showed close agreement with the commercial serum and milk IgG assay data. The level of IgG in saliva was notably lower in comparison to IgA. The newly developed method exhibits the potential to serve as an easily transferable tool for epidemiological studies.

**Electronic supplementary material:**

The online version of this article (doi:10.1186/s12917-015-0552-0) contains supplementary material, which is available to authorized users.

## Background

Schmallenberg virus (SBV), which emerged recently in Europe, was first reported in Germany in a farm near the town of Schmallenberg in late 2011 [1]. Metagenomic analysis identified a novel *Bunyaviridae* family *Orthobunyavirus*, which subsequently was isolated from blood specimens of infected animals. Recent analysis revealed that SBV is most closely related to the Douglas and Sathuperi viruses belonging to the Simbu serogroup in *Orthobunyavirus* [2]. In cattle, clinical symptoms include fever, loss of appetite, reduced milk yield and diarrhoea. Also, SBV infection has been implicated in many cases of severely malformed bovine and ovine offspring [3–8]. Quantitative reverse transcriptase PCR (q-PCR) is the primary diagnostic assay developed by laboratories in affected countries [1]. This assay has limitations in detecting infected individuals based on blood samples, as it only detects viral RNA when the bovine is viraemic [9]. The first indirect enzyme-linked immunosorbent assay (ELISA) to detect SBV-specific antibodies in serum and milk samples became commercially available shortly after the emergence of SBV (SBV indirect ELISA, IDvet, France) [10, 11]. Testing of bulk tank milk samples by ELISA has been advocated as a convenient way to determine herd-level exposure to SBV [12, 13].

With the availability of vaccines against SBV, it has become important to test animals and apply the test results for herd management. For example a positive bulk tank milk sample indicates that herd-level vaccination is not necessary as natural immunity is present. This test is suitable for dairy farms, but not for males or young cattle. Saliva has a number of advantages over serum for diagnosis. Saliva collection is cheap, non-invasive, is easy to store and transport. Currently a commercial saliva based test for SBV-sero-testing is not available [14, 15].

The aim of this study was to compare the antibody levels in sera, milk and saliva and to develop a method for the detection of SBV-specific antibodies in the saliva of cattle.

## Methods

### Recombinant SBV N antigen

The cloning of the SBV N gene and purification under denaturing conditions of the recombinant SBV N antigen was recently described [16]. Our recent findings indicated that the SBV N protein purified under native conditions exhibited better antigenicity than the SBV N protein purified under denaturing conditions (See Additional file 1: Figure S1 and Additional file 2: Figure S2). Higher OD readings with positive sera could be obtained using SBV N protein purified under native conditions (See Additional file 2: Figure S2). Thus, for the development of new diagnostic kits, the SBV N protein purified under native conditions seems to have better antigenic characteristics and sero-diagnostic potential. *Saccharomyces cerevisiae* AH22-214 yeast transformation with the plasmid vector was described previously [16, 17]. In this study the pFGG-SBV-N plasmid vector, containing non-tagged full length SBV N protein coding sequence, was used for transformation. After the transformation yeast cells were inoculated in 500 ml of YEPD growth medium supplemented with 5 mM formaldehyde and grown with shaking at 30 °C for 24 h. 500 ml of YEPG induction medium (yeast extract 1 %, peptone 2 %, galactose 5 % supplemented with 5 mM formaldehyde) was added and the yeast cells were grown for an additional 17 h. The cells were harvested, washed with distilled water and frozen at -20 °C until further use. Thawed cells were suspended in 34 ml of PBS at pH 7.4 and 34 g of glass beads (0.5 mm diameter, Sigma-Aldrich Co., St. Louis, MO, USA) were added. Cells were disrupted mechanically by vortexing at 4 °C for 7 min. The lysates were cleared from debris by centrifugation at 2000 × g for 3 min. The insoluble protein fraction was separated by centrifugation at 10,000 × g for 30 min at 4 °C. The supernatant was collected and placed on top of a 70 %/60 %/50 %/40 % sucrose gradient in PBS in an ultracentrifuge tube. The proteins were centrifuged at 110,396 g for 15 h. The 70 % and 60 % gradients, containing the SBV N protein, were collected and concentrated by centrifugation for 2 h at 37,000 rpm through 30 % sucrose solution. The protein pellet, containing the SBV N protein was suspended in 2 ml of PBS. The purity of the resultant N protein was more than 90 % as suggested by SDS-PAGE (See Additional file 1: Figure S1). Two ml of glycerol were added and the protein was kept at -20 °C until further use.

### Sera, milk and saliva collection

Bovine blood, milk and saliva samples were collected in September 2014 from dairy farms in different regions in Lithuania. All bovines were clinically healthy at the time of sampling. Samples were stored at -20 °C until tested. Saliva specimens were collected using the Copan Flocked Swabs (Copan, Brescia, Italy) device according to the manufacturer’s instructions. The study was conducted according to the Law on the Care, Keeping and Use of Animals, No. 8-500 of the Republic of Lithuania. This research does not need to be approved by an appropriate ethics committee. This is not field studies or experimental research on animals and it complies with institutional, national, or international guidelines.

The sera were tested for antibodies against SBV using a commercially available ELISA kit (ID Screen Schmallenberg virus Indirect, IDvet, Grabels, France) [10] before testing with indirect ELISA with the recombinant SBV N protein.

Reference sera, certified negative or positive for SBV-specific antibodies, were kindly provided by Dr. H. Schirrmeier (Friedrich Loeffter Institut, Germany; [18]).

### Indirect IgG ELISA for detection of the SBV N protein-specific antibodies in bovine serum

The ELISA was carried out as described by Lazutka et al. [16].

### Indirect IgG ELISA for the detection of SBV N protein-specific antibodies in cow’s milk and saliva, and indirect IgA ELISA for saliva

The following protocol was adopted after optimization of the assays. Microtiter plates (Nerbe Plus GmbH, Winsen/Luhe, Germany) were coated with 2 μg/ml of recombinant SBV N protein in 100 μl of 0.05 M carbonate-bicarbonate coating buffer (pH9.6) and incubated overnight at 4 °C. Plates were washed three times with PBST (PBS and 1 % Tween-20) and then blocked by the addition of 150 μl of blocking buffer per well (1x Roti-Block, Carl Roth GmbH & Co, Karlsruhe, Germany). The plates were incubated at room temperature for 1 h. After blocking, the plates were washed three times with PBST and 100 μl aliquots of milk specimens, diluted 1:10 in PBST, and were added to the wells. Plates were incubated for 1 h at 37 °C and washed five times with PBST. One hundred μl aliquots of rabbit anti-bovine IgG (Sigma-Aldrich Co., St. Louis, MO, USA) conjugated to HRP, diluted 1:20,000 (v/v) in PBST, were added to each well and the plates were incubated for 1 h at 37 °C. After washing five times with PBST, 100 μl of TMB substrate (Invitrogen, Frederick, USA) was added to each well and the enzyme reaction was stopped with an equal volume of 1 M H_2_SO_4_ solution, after 10 min. of incubation. The optical density at 450 nm was determined for each sample using an ELISA plate reader (Sunrise Tecan, Mannedorf, Switzerland).

The protocol was similar for saliva anti-SBV IgA ELISA, except saliva samples were diluted 1:3 in PBST and sheep anti-bovine IgA, (AbD Serotec, Biorad, Kidlington, UK) conjugated to HRP, were diluted 1:20,000 in PBST with 5 % RotiBlock and 1 % chicken serum (Gibco/Invitrogen, Paisley, UK). For the saliva anti-SBV IgG ELISA 4 μg/ml of the SBV N protein was used per well. Nunc Maxisorp microtiter plates (Thermo Scientific, Roskilde, Denmark) were blocked with 150 μl of 5 % chicken serum in PBS per well. Saliva samples were diluted 1:3 and rabbit anti-bovine Fab’2 IgG (LifeSpan BioSciences, Inc., Seattle WA, USA) conjugated to HRP were diluted 1:30,000 in PBST with 5 % Roti-Block and 1 % chicken serum.

### Determining the cut-off values of the different ELISAs

The cut-off value of our serum anti-SBV IgG system was calculated as follows: 27 serum samples that were negative according to the commercial indirect SBV ELISA test were tested against the SBV N protein. The optical density of a sample was divided by the optical density of a reference serum [18] sample and a sample-to-positive (S/P) value in percent was obtained for each sample. The calculation of S/P values was performed according to Breard et al. [10]. The cut-off value for the serum IgG system was then determined using the following formula:

$$ \mathrm{Cut}\ \mathrm{off}\kern0.5em =\kern0.5em \left({\mathrm{x}}_1 + {\mathrm{x}}_2 + \dots + {\mathrm{x}}_{\mathrm{n}}\right)/\mathrm{n} + 3\ *\ \mathrm{S}\mathrm{D}\left({\mathrm{x}}_1,\ {\mathrm{x}}_2, \dots,\ {\mathrm{x}}_{\mathrm{n}}\right) $$

where *x* is an S/P value of the individual sample, *n* is the number of samples and *SD* is the standard deviation of the set of samples. The samples that showed reactivity within the average plus 2*SD and average plus 3*SD range were considered doubtful.

We did not have enough negative milk or saliva samples available for us to determine the cut-off values for our milk and saliva assays. Instead, S/P values were calculated after incubating milk or saliva samples with the closely related hantavirus Andes nucleocapsid protein [19]. Other authors also used different antigens for determination of cut-off values. Starkey and co-authors [20] report the use of glutathione *S*-transferase (GST) tagged proteins in enzyme immunoassays (EIAs). They have used GST as a control antigen to permit estimation of background OD in EIAs. Lin and co-authors [21] used a bovine bocavirus protein as a control antigen to define the cut-off in ELISAs established to detect human bocavirus specific antibodies.

The S/P cut-off values for milk IgG, serum IgG, saliva IgA and saliva IgG were 30 %, 28 %, 15 % and 10.5 % accordingly. The cut-off values for these systems were determined as described above.

### Evaluation of diagnostic assays in different specimen types

As a first step towards the development of an SBV N ELISA, checkerboard titrations were performed to determine the optimal concentration of the SBV N antigen. To optimize the plate coating, the recombinant SBV N protein was immobilized on ELISA plates at four different concentrations: 4 μg/ml; 2 μg/ml; 1 μg/ml; 0.5 μg/ml. To determine the optimal milk sample dilution, milk samples were serially diluted ranging from 1:5 to 1:50. Saliva samples were tested at dilutions ranging from 1:2 to 1:128. The anti-bovine IgG conjugate was diluted from 1:10,000 to 1:80,000 and the anti-bovine IgA-HRP conjugate was diluted from 1:5000 to 1:40,000. The three milk and saliva samples that were used for the evaluation of the assay had matching serum samples that were characterized by the commercial test as a strong positive, weak positive and negative. After the optimization the tests were used to analyze bovine sera, milk and saliva samples.

### Statistical analysis

Statistical significance between separate tests was calculated using MedCalc and Microsoft Excel 2007 software.

## Results

### Optimization of the assays

The optimal antigen concentration for plate coating as determined by a checkerboard titration was 2 μg/ml for milk IgG and saliva IgA assays and 4 μg/ml for saliva IgG assay. Positive samples revealed significantly higher OD values (0.6 – 2.8) with SBV N protein as compared to the control hantavirus Andes N antigen (0.03–0.15). It was decided to use a dilution of 1:10 for the milk specimens and a saliva dilution of 1:3 as these dilutions gave good discrimination between positive and negative reactions and were more economical in the use of the sample. Nerbe plus plates were used for milk IgG and saliva IgA assays, but for saliva IgG assay we have chosen Nunc Maxisorp plate, in order to increase the sensitivity of the assays. The maximum reactivity was achieved using weak positive samples, when Nunc Maxisorp plates were used for antigen coating. Nerbe plus plates were not sufficient to detect specific IgG in saliva with low antibody titers, therefore Nunc Maxisorp plate was used to compensate low IgG levels in saliva. A 1:30,000 dilution of anti-bovine IgG -HRP conjugate, and 1:20,000 dilution of anti-bovine IgA-HRP conjugate gave the greatest discrimination between reactivity of positive samples with SBV N antigen and the control antigen - hantavirus Andes N protein.

The saliva sample and anti-bovine IgG/IgA conjugates were diluted in PBS with 5 % Roti-block and 1 % chicken serum solution. The solution was found to be more effective in blocking non-specific binding, leading to lower OD values for the negative control and therefore higher *S*/*P* ratios.

### Comparison of SBV specific antibody response in milk and saliva samples

Using the newly developed indirect IgG and IgA ELISAs we analyzed sera and their matched milk and saliva samples from 58 randomly selected dairy cows that had been tested before with commercial serum IgG and milk IgG assays. 54 serum samples were positive, one sample was negative and three samples were doubtful in commercial assay. The doubtful serum samples were excluded from further examination. Fifty-two out of 54 positive serum samples were also positive in our developed assay, while two samples were doubtful. One sample was negative in both assays. The agreement between the two tests for the serum IgG assays was 96.30 % (95 % Confidence Interval: 87.25 % - 99.55 %). A small correlation of 0.14 (*p =* 0.00515) between the optical densities of the same serum samples in indirect IgG SBV ELISA and in commercial ELISA was observed.

Further, 58 milk samples were screened with commercial IDvet IgG ELISA test. Fifty-one positive and one negative milk sample was determined by commercial assay. 6 milk samples were determined as doubtful and were excluded from further examination. The remaining 52 samples were tested with newly developed indirect IgG ELISA test. Forty-eight samples were described as positive and one sample was negative. 3 samples, which were determined as positive in commercial test, were doubtful in our test. All 48 positive milk samples in our assay were also positive in commercial assay and the negative sample in our assay was also negative in commercial assay. Our indirect milk IgG assay achieved a sensitivity of 94.12 % (95 % CI: 83.76 % - 98.77 %) (Table 1-A). Based on these values 98 % cows were SBV seropositive. The reproducibility of independently performed ELISAs was high (R^2^ = 0.93). The correlation between the OD of milk samples in commercial and in our developed assay was low (R^2^ = 0.15, *p =* 0.00597).

**Table 1:** Comparison of bovine serum, milk and saliva samples analyzed with newly developed indirect SBV N ELISA tests with commercial IDvet serum or milk indirect IgG ELISAs

(A)				
		IDvet milk indirect ELISA		
		Positive	Negative	Total
Indirect milk IgG ELISA	Positive	48	0	48
	Negative	3	1	4
	Total	51	1	52
(B)				
		IDvet serum indirect ELISA		
		Positive	Negative	Total
Indirect saliva IgG ELISA	Positive	46	0	46
	Negative	8	1	9
	Total	54	1	55
(C)				
		IDvet serum indirect ELISA		
		Positive	Negative	Total
Indirect saliva IgA ELISA	Positive	53	0	53
	Negative	1	1	2
	Total	54	1	55
(D)				
		Indirect saliva IgA ELISA		
		Positive	Negative	Total
Indirect saliva IgG ELISA	Positive	45	1	46
	Negative	8	1	9
	Total	53	2	55

Comparison of commercial milk ELISA with our developed indirect milk IgG ELISA (A), commercial serum ELISA with our indirect saliva IgG (B) and saliva IgA (C) ELISA, between saliva IgG and IgA ELISAs (D)

**Fig. 1:**
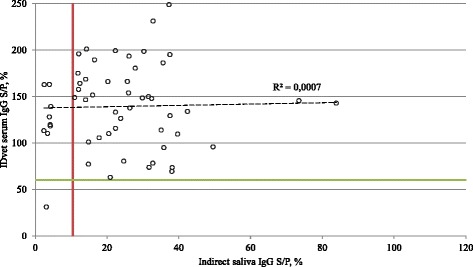
Relationship between S/P ratios of individual saliva and serum samples. Saliva samples from dairy cows were tested with indirect IgG ELISA using recombinant SBV N protein and serum samples were tested with commercial IgG IDvet ELISA. The horizontal line represent the cut-off value of serum IgG IDvet ELISA, while the vertical line represent the cut-off value of saliva IgG test. Dashed line marks linear regression, *R* squared is the coefficient of determination

**Fig. 2:**
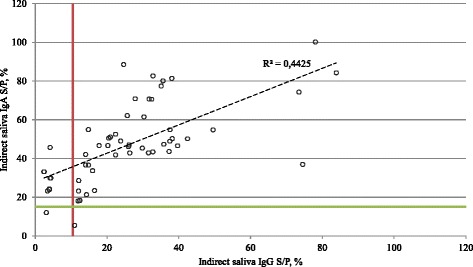
Relationship between S/P ratios of indirect saliva IgG and IgA assays. Saliva samples from dairy cows were tested with indirect IgG and IgA ELISAs using recombinant SBV N protein. The horizontal line represent the cut-off value of saliva IgA test, while the vertical line represent the cut-off value of saliva IgG test. Dashed line marks linear regression, *R* squared is the coefficient of determination

We then tested if the 55 animals tested for anti-SBV IgG in their serum contain SBV-specific IgG and IgA antibodies in their matched saliva samples. Forty-six individuals were positive for SBV IgG antibodies in saliva. Eight cows with no antibodies in saliva had mid-level antibody titers in their sera (Table 1-B). The IgG antibody titer was lower in the saliva than in serum in all cows tested. The only sample that was negative in serum assay was also negative in saliva assay. Thus the sensitivity and specificity was 85.19 % (95 % CI: 72.88 % - 93.38 %) and 100 % (95 % CI: 2.5 % -100 %), respectively, compared to the commercial IgG assay. A comparison of saliva IgG and serum IgG pairs is shown in Fig. 1. Fifty-three cows tested positive in saliva IgA test compared to 54 positive cows in IDvet IgG test. One cow tested negative in both serum IgG and saliva IgA assays and one saliva sample was described as false-negative (Table 1-C). The sensitivity of saliva IgA ELISA was 98.15 % (95 % CI: 90.11 % - 99.95 %). Forty-five saliva samples were positive for the presence of SBV-specific IgG’s out of 53 saliva IgA positives. Eight saliva samples displayed reactivity below the cut-off value in IgG assay while showing a positive reaction in an anti-SBV IgA ELISA (Fig. 2). One saliva IgG positive sample was negative in saliva IgA assay, while one sample was negative in both assays (Table 1-D, Fig. 2). The sensitivity of the saliva IgG test compared to saliva IgA test is 84.91 % (95 % CI: 72.41 % - 93.25 %). There was no linear correlation (R^2^ = 0.0007) for saliva/serum IgG pairs (Fig. 1). Linear regression analysis estimated the coefficient of determination (R^2^) between antibody concentration in saliva IgG and saliva IgA at 0.44 with *p* value of 2.24 * 10^-8^ (Fig. 2).

## Discussion

A commercially available antibody ELISA was recently evaluated [11, 13] to detect SBV-specific antibodies in serum and milk samples. Our study aimed to evaluate antibody screening assays for use on both individual milk and saliva samples. We did this by comparing the results of individual milk and saliva antibody testing to individual serum antibody testing. Milk and saliva sample collection is cheap, noninvasive and animal welfare-friendly. However milk and saliva samples are not sterile and are subject to bacterial degradation over time. Furthermore, salivary composition is influenced by the method of collection and the degree of stimulation of salivary flow. Stimulation of salivation before sample collection may lower antibodies concentration in saliva. Dilution effect of fluids from the salivary glands requires extremely sensitive tests that are able to detect small quantities of antibody. Saliva contains antibodies in concentrations that are 1000-fold less than those in blood. Sensitive detection systems are thus needed to reveal the utility of saliva as a diagnostic medium [14, 22].

Development of an assay for detection of antibodies in milk and saliva required optimization of numerous parameters to maximize sensitivity and specificity. Initial experiments optimized the concentration of SBV N protein used as coating antigen, selection of the microtest plate, the dilution of the test milk and saliva, and the dilution of the HRP conjugated secondary antibody to provide the best discrimination between known positive and negative specimens as determined by commercial test with adequate serum sample.

Three serum samples and six milk samples were excluded from the study for being doubtful. Some authors suggest repeating the tests with longer incubation times to define the antibody status of doubtful samples, or they exclude these samples from their investigation [23, 24]. As the several samples taken over period of time from the same bovine, were not available for the study, therefore the doubtful samples were excluded from further study.

There was close agreement between matched serum and milk IgG assays. The sensitivity of 96 % for serum IgG assay compared to commercial serum IgG ELISA was achieved. This shows a slight improvement over our recently developed indirect serum anti-SBV IgG ELISA [16], where the sensitivity of the assay was 95 %. A low degree of correlation between optical densities of the same serum samples in commercial and our tests was observed and may suggest that different epitopes on the nucleocapsid protein are recognized in these assays.

These results promoted the idea that our newly purified recombinant SBV N protein could also be used to detect antibodies against SBV in milk or saliva samples. All our developed assays agreed with the commercial ones. The agreement of our newly developed milk IgG assay with the commercial test was 94 %. Detection of SBV-specific IgA in saliva yielded similar results, with 98 % sensitivity. However the saliva IgG assay is less sensitive than the saliva IgA assay, probably because of a lower IgG concentration in saliva [25].

After SBV infection antibodies against SBV can be found in bovine serum, milk and saliva. The relationship between individual serum and milk antibodies for SBV was established by others [12]. We suggest using non-invasive sample extraction methods when possible, as antibodies against SBV are present in milk and saliva.

We show that IgA is a reliable marker for SBV diagnosis using bovine saliva samples. There are only few studies demonstrating the production of detectable levels of viral-specific antibodies in bovine saliva samples. This is the first description of a diagnostic assay to SBV based on saliva samples. To date most research on cattle saliva as a diagnostic sample has been carried out with regard to foot and mouth disease virus (FMDV) diagnostics [26, 27].

## Conclusions

We have shown that milk and saliva samples are suitable substitutes for serum with minimal loss of sensitivity. Therefore, milk and saliva specimens have the potential to replace serum based screening in large-scale seroprevalence studies.

## Additional files

Additional file 1: Figure S1. Analysis of yeast cell lysates and purified SBV N proteins by Western Blot (A) and SDS-PAGE (B). M – Spectra Broad Range Prestained Protein Ladder (Thermo Fisher Scientific Baltics, Vilnius, Lithuania), 1 – lysate of *S. cerevisiae* yeast transformed with mock pFGG plasmid, 2 – 6His- SBV N protein after nickel-affinity chromatography purification under denaturing conditions, 3– SBV N protein after ultracentrifugation in sucrose gradient. Western blotting was performed using mouse monoclonal antibodies raised against SBV N, Mab code 8G10 [16]. (PDF 1256 kb) Additional file 2: Figure S2. Comparison of the reference sera reactivity with various antigens using IgG ELISA. Positive, weak positive and negative bovine reference sera [18] were tested. Columns represent antibody response against SBV N antigen purified under native conditions (black colums), 6His-SBV N purified under denaturing conditions [16] (grey columns) and control hantavirus Andes N antigen [19] (white columns). The OD values are expressed as obtained in arbitrary units. Bars indicate average values plus standard deviation. (PDF 187 kb)

## Abbreviations

ELISA: Enzyme linked immunosorbent assay
SBV: Schmallenberg virus
SBV N: Schmallenberg virus nucleocapsid
HRP: Horseradish peroxidase
S/P: Sample-to-positive value in percent.
