# Supplementary figures and images for: Saliva as an alternative specimen for detection of Schmallenberg virus-specific antibodies in bovines
Source: BMC Vet Res. 2015 Sep 15;11:237. doi: 10.1186/s12917-015-0552-0 (PMC4570040; doi:10.1186/s12917-015-0552-0)

(A)

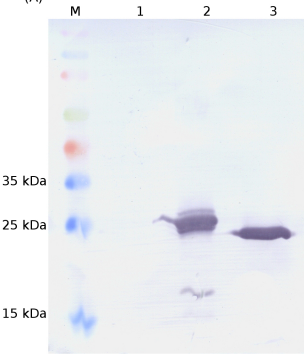

(B)

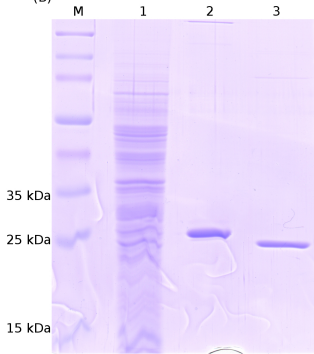

Supplement: Additional file 1: Figure S1. — Analysis of yeast cell lysates and purified SBV N proteins by Western Blot (A) and SDS-PAGE (B). M – Spectra Broad Range Prestained Protein Ladder (Thermo Fisher Scientific Baltics, Vilnius, Lithuania), 1 – lysate of S. cerevisiae yeast transformed with mock pFGG plasmid, 2 – 6His- SBV N protein after nickel-affinity chromatography purification under denaturing conditions, 3– SBV N protein after ultracentrifugation in sucrose gradient. Western blotting was performed using mouse monoclonal antibodies raised against SBV N, Mab code 8G10 [16]. (PDF 1256 kb) [file 12917_2015_552_MOESM1_ESM.pdf]

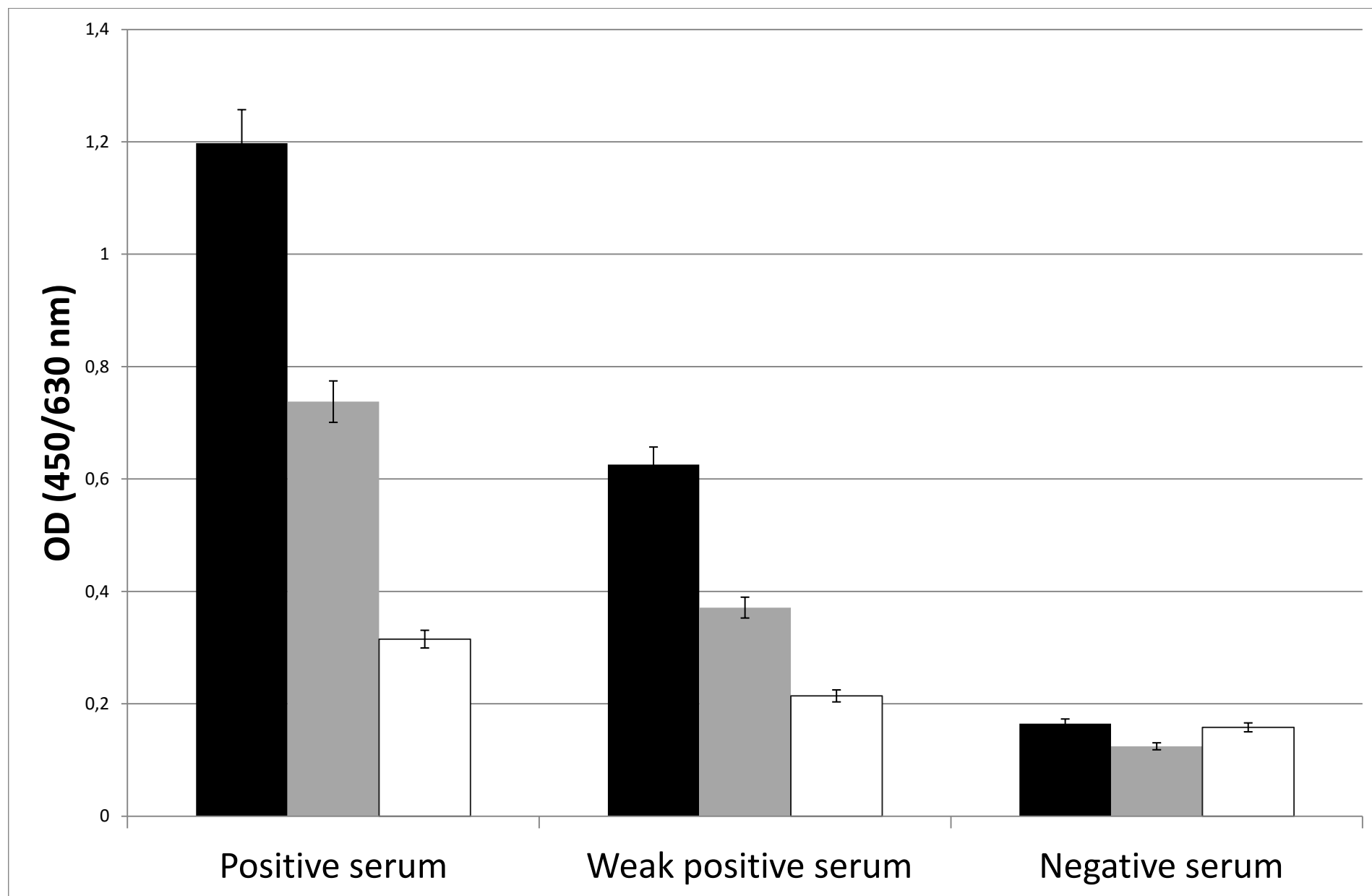

Supplement: Additional file 2: Figure S2. — Comparison of the reference sera reactivity with various antigens using IgG ELISA. Positive, weak positive and negative bovine reference sera [18] were tested. Columns represent antibody response against SBV N antigen purified under native conditions (black colums), 6His-SBV N purified under denaturing conditions [16] (grey columns) and control hantavirus Andes N antigen [19] (white columns). The OD values are expressed as obtained in arbitrary units. Bars indicate average values plus standard deviation. (PDF 187 kb) [file 12917_2015_552_MOESM2_ESM.pdf]
